# Deep Learning Cluster Structures for Management Decisions: The Digital CEO †

**DOI:** 10.3390/s18103327

**Published:** 2018-10-04

**Authors:** Will Serrano

**Affiliations:** Intelligent Systems and Networks Group; Imperial College London, London SW7 2AZ, UK; g.serrano11@imperial.ac.uk

**Keywords:** random neural network, deep learning clusters, cognitive packet network, quality of service, cybersecurity, routing

## Abstract

This paper presents a Deep Learning (DL) Cluster Structure for Management Decisions that emulates the way the brain learns and makes choices by combining different learning algorithms. The proposed model is based on the Random Neural Network (RNN) Reinforcement Learning for fast local decisions and Deep Learning for long-term memory. The Deep Learning Cluster Structure has been applied in the Cognitive Packet Network (CPN) for routing decisions based on Quality of Service (QoS) metrics (Delay, Loss and Bandwidth) and Cyber Security keys (User, Packet and Node) which includes a layer of DL management clusters (QoS, Cyber and CEO) that take the final routing decision based on the inputs from the DL QoS clusters and RNN Reinforcement Learning algorithm. The model has been validated under different network sizes and scenarios. The simulation results are promising; the presented DL Cluster management structure as a mechanism to transmit, learn and make packet routing decisions is a step closer to emulate the way the brain transmits information, learns the environment and takes decisions.

## 1. Introduction

Our brain takes decisions in a structured way while performing several functions at the same time. Our brain learns about the environment from our five senses; it stores memories to preserve our identity; it makes judgements on different situations; it protects itself against external threats or attacks. Our brain is formed by clusters of neurons [1] specialized in learning from different senses where information is transmitted as positive and negative spikes or impulses. It functions with two types of memories [2]; short-term memory is used for quick decisions and task-related actions whereas long-term memory preserves our identity and security. Another brain duality consists of its two operation modes [3]; consciousness under normal activities and unconsciousness under emergency situations such as being under external attack or routine operations like storing information while sleeping.

This paper presents the association of the most complex biological system; our brain with the most complex artificial system represented in large data networks: The Internet; the information infrastructure of the Big Data and the Web. The link between both of them is the Random Neural Network (RNN). Data networks collect information from users and transmit it to different locations; to perform this activity, they are required to make routing decisions based on different Quality of Service metrics while storing routing tables in memory under the threat of Cyber-attacks.

This paper proposes a Deep Learning (DL) Cluster Structure for Management Decisions that emulates the way the brain learns and makes choices and combines different Learning Algorithms. The proposed model combines the Random Neural Network Reinforcement Learning for fast local decisions and DL for long-term memory to remember network identity: QoS metrics (Delay, Loss and Bandwidth) and Cyber keys (User, Packet and Node). In addition, this paper includes a layer of DL management clusters (QoS, Cyber and CEO) that take the final routing decision based on the inputs from the DL QoS clusters and RNN Reinforcement Learning algorithm.

The Deep Learning Cluster Structures has been applied in the Cognitive Packet Network (CPN) for Quality of Service metrics and Cyber Security keys in Management Decisions based on packet routing and flow control. The RNN Reinforcement Learning Algorithm is chosen under normal or conscious operations due to its fast and adaptable routing learning as short memory whereas DL clusters are selected under external cyber-attacks. Deep Learning clusters take routing decisions based on the long-term memory in unconsciousness operation as safe and resilient, although inefficient and inflexible, routing.

A concepts review of Cybersecurity, Deep Learning and Deep Reinforcement Learning with their associated literature research is described in Section 2. The mathematical model of the Deep Learning clusters Structures for management decisions is defined in Section 3. The implementation of the QoS, Cyber and Management Clusters is presented on Section 4. The validation of the proposed model under different QoS and Cyber scenarios in small (nine nodes, one decision layer), medium (16 nodes, two decision layers) and large (25 nodes, three decision layers) is described in Section 5. Final discussion and bibliography are shared in Section 6 and References respectively.

## 2. Research Background

### 2.1. Cybersecurity

The expansion of the connectivity provided by the Ethernet and Internet protocols has enabled new industrial, technological and social applications and services, however, users are increasingly under new cybersecurity threats and risks. Ericsson [4] introduces cybersecurity issues and threats within Power Communications Systems in a smart grid infrastructure where network vulnerabilities and information security domains are analyzed. Ten [5] presented a survey on cybersecurity of critical infrastructure; in addition, they propose a Supervisory Control And Data Acquisition (SCADA) framework based on four procedures: Real-time monitoring, anomaly detection, impact analysis and mitigation strategy. They model an attack tree analysis with an algorithm for cybersecurity evaluation that incorporates password policies and port auditing. Cruz et al. [6] presented a distributed intrusion detection system for SCADA systems that includes different types of security agents tuned for each specific domain: Development of a network, device and process level capabilities, integration of signature and anomaly-based techniques against threats and finally the adoption of a distributed multi-layered design with message queues to transmit predefined events between elements. Wang et al. [7] proposed a framework to facilitate the development of adversary resistant Deep Neural Networks (DNN) by inserting a data transformation module between the sample and the DNN that avoids threat samples with a minimum impact on the classification accuracy. Tuor et al. [8] presented an unsupervised Deep Learning approach to detect anomalous network activity from system logs in real-time where events are extracted as features and the DNN learns users’ normal behavior or anomaly as potential malicious behavior. Wu et al. [9] presented a classification of cyber-physical attacks and risks in cyber manufacturing systems with possible mitigation measures such as supervised machine learning for classification and unsupervised machine learning for anomaly detection on physical data. Kim et al. [10] proposed a new cyber defensive computer control system architecture based on the diversification of hardware systems and unidirectional communications assuming that the detection and prevention of cyber-attacks will never be complete.

### 2.2. Deep Learning

Deep Learning is characterized by using a cascade of l-layers of non-linear processing units for feature extraction and transformation; each successive layer uses the output from the previous layer as input. Deep Learning learns multiple layers of representations that correspond to different levels of abstractions; those levels form a hierarchy of concepts where the higher the level, the more abstract concepts are learned. Schmidhuber et al. [11] examined DL in neural networks; the work includes deep supervised learning, unsupervised learning, reinforcement learning and evolutionary computation. It also includes an indirect search for short programs encoding deep and large networks. The success of machine learning algorithms generally depends on data representation. In order to obtain the appropriate objectives for learning good representations, computing representations and the geometrical connections between representation learning, density estimation and manifold learning; Bengio et al. [12] reviewed recent work in the area of unsupervised feature learning and DL, which includes advances in probabilistic models. They proposed a new probabilistic framework to include likelihood based probabilistic models, reconstruction based models such as autoencoder variants and geometrically based manifold learning approaches. Jie et al. [13] proposed a progressive framework to deep optimize neural networks. They combine the stability of linear methods with the ability of learning complex and abstract internal representations of DL methods. They introduce a linear loss layer between the input layer and the first hidden non-linear layer of a traditional deep learning model where the loss objective for optimization is a weighted sum of linear loss of the added new layer and non-linear loss of the last output layer.

The predominant algorithm to train DL uses stochastic gradient descent methods, although they are easy to implement, gradient descent is difficult to tune and parallelize. In order to overcome this issue, Le et al. [14] studied the advantages and disadvantages of off-the-shelf optimization algorithms in the context of simplification and to speed up the process of pre-training the unsupervised feature learning. Deep networks have been successfully applied to unsupervised feature learning for single modalities such as text, images or audio. However, Ngiam, J. et al. [15] proposed an application of deep networks to learn features over multiple modalities to demonstrate that cross-modality feature learning performs better than single modality learning. The deep network is trained with audio only data but tested with video only data and vice versa. Deep Neural Networks (DDNs) provide good results when large labeled training sets are available, however, they perform worse when mapping sequences to sequences. In order to address this issue, Sutskever et al. [16] presented an approach to sequence learning that makes minimal assumptions on the sequence structure. They use a multilayered Long Short-Term Memory (LSTM) to map the input sequence to a vector of a fixed dimensionality, and then another deep LSTM to decode the target sequence from the vector. Bekker et al. [17] proposed an intracluster training strategy for DL with applications to language identification where the language clusters are used to define a cost function to train a neural network. Their method trains a classifier and analyzes the obtained confusion matrix where languages are simultaneously clustered in the columns and the rows of the confusion matrix. The language clusters are then used to define a modified cost function for training a neural network that learns to distinguish between the true language and languages within the same cluster.

### 2.3. Deep Reinforcement Learning

Deep Learning enables Reinforcement Learning to scale decision-making solutions that were previously unmanageable. A new algorithm called Double Deep Q Network (DQN) that generalizes an arbitrary function approximation was proposed by Hasselt et al. [18]. The algorithm includes DNN and reduces overestimations by decomposing the max operation in the target into action selection and action evaluation. Although DQN solves problems with high dimensional observation spaces; it can only manage discrete and low-dimensional action spaces. As presented by Lillicrap et al. [19], DQN depends on finding the action that maximizes the action-value function which in the continuous-valued case requires an iterative optimization process at each step. In order to overcome this issue, they propose an algorithm based on the deterministic policy gradient that can operate over continuous spaces. A framework for Deep Reinforcement Learning (DRL) that asynchronously executes multiple agents in parallel on multiple instances of the environment is proposed by Mnih et al. [20]. This parallelism decorrelates the agent’s data into a more stationary process using gradient descent for optimization of deep neural network controllers. A neural network architecture for model-free reinforcement learning where a dual network represents two separate estimators: one for the state value function and the other for the state-dependent action advantage function is presented by Wang et al. [21]. The two streams are combined via a special aggregating layer to produce an estimate of the state action-value function. A benchmark for continuous simple actions, high state and action dimensionality control, tasks with partial observations and tasks with a hierarchical structure is presented by Duan et al. [22]. They divide 31 tasks into basic control, locomotion and partially observable in order to achieve higher hierarchical structure tasks where higher level decisions can reuse lower level skills. Challenges posed by reproducibility, experimental techniques, and reporting procedures of DRL methods is investigated by Henderson et al. [23]. They present the variability in reported metrics and results when comparing against common baselines and suggest guidelines to make future results in Deep RL more reproducible. DRL for resource management problems in systems and networking is applied by Mao et al. [24]. The decision-making tasks where appropriate taken solutions depend on understanding the workload and environment experience.

## 3. Deep Learning Cluster Structures for Management Decisions

### 3.1. The Random Neural Network—Reinforcement Learning

**Figure 1:**
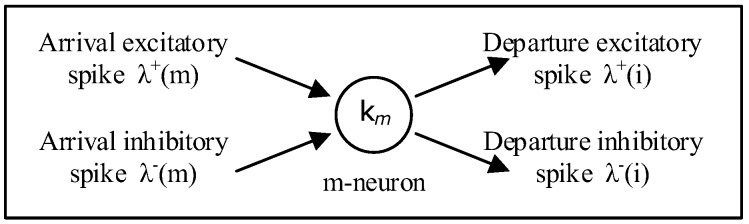
The Random Neural Network (RNN).

The Random Neural Network (RNN) [25,26,27] represents more closely how signals are transmitted in many biological neural networks where they travel as spikes or impulses, rather than as analogue signal levels (Figure 1). The RNN is a spiking recurrent stochastic model for neural networks. Its main analytical properties are the “product form” and the existence of the unique network steady-state solution. It has been applied in different applications including search for exit routes for evacuees in emergency situations [28,29], pattern-based search for specific objects [30], video compression [31], and image texture learning and generation [32].

The RNN is composed of *M* neurons each of which receives excitatory (positive) and inhibitory (negative) spike signals from external sources which may be sensory sources or neurons (Figure 1). These spike signals occur following independent Poisson processes of rates *λ*^+^(*m*) for the excitatory spike signal and *λ*^−^(*m*) for the inhibitory spike signal respectively, to cell *m* Є {1, …, *M*}.

The RL algorithm is based on the RNN with at least as many nodes as the number of decisions to be taken is generated where neurons are numbered 1, …, *j*, …, *n*; therefore for any decision *i*, there is some neuron *i*. Decisions in this RL algorithm with the RNN are taken by selecting the decision *j* for which the corresponding neuron is the most excited, the one with has the largest value of *q_j_*. The state *q_j_* is the probability that it is excited, these quantities satisfy the system of non-linear equations:

(1) $$q_{j}=\frac{\lambda^{+}(j)}{r\left(j\right){+\lambda }^{-}(j)}.$$

### 3.2. The Cognitive Packet Network

**Figure 2:**
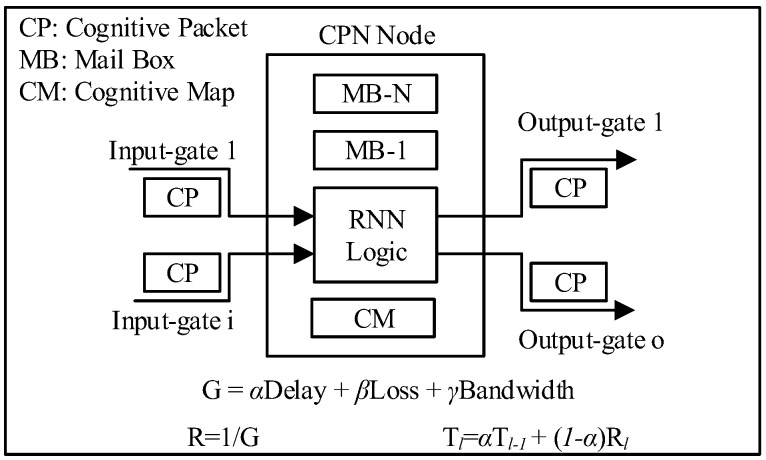
The Cognitive Packet Network (CPN).

The CPN was introduced by Gelenbe et al. [33,34,35,36,37]; it has been tested in large-scale networks up to 100 nodes with worst and best case performance scenarios. The CPN assigns routing and flow control capabilities to the packets rather than the nodes (Figure 2). QoS goals are assigned to Cognitive Packets (CP) within the CPN, which they follow when making routing decisions themselves with minimum dependence on the nodes. Cognitive Packets learn from experience of other CP packets with whom they interchange network information using n Mailboxes (MB) and their own inspection about the network storing network information in their Cognitive Map (CM).

Given some Goal G that the agent has to achieve as a function to be to be optimized and reward *R* as a consequence of the interaction with the environment; successive measured values of the *R* are denoted by *R_l_*, *l* = 1, 2, … these are used to compute a decision threshold:

(2) $$T_{l}{=\alpha T}_{l-1}{+(1-\alpha )R}_{l},$$

where *α* is some constant 0 < *α* < 1. The agent takes the lth decision which corresponds to neuron *j* and then the *l*_th_ reward *R_l_* is measured and its associated *T_l−1_* is calculated.

### 3.3. Deep Learning Clusters

**Figure 3:**
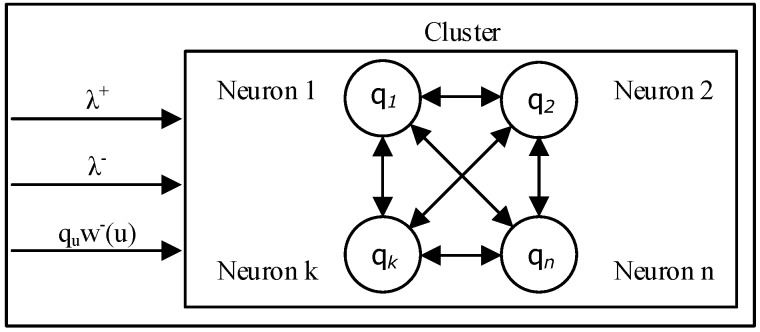
Cluster of Neurons.

Deep Learning Clusters with RNN is described by Gelenbe, E. and Yin, Y. [38,39]. This model is based on the generalized queuing networks with triggered customer movement (G-networks) where customers are either “positive” or “negative” and customers can be moved from queues or leave the network (Figure 3). G-Networks are introduced by Gelenbe et al. [40,41]; an extension to this model is developed by Gelenbe et al. [42] where synchronized interactions of two queues could add a customer in a third queue. The model considers a special network *M*(*n*) that contains *n* identically connected neurons, each which has a firing rate *r* and external inhibitory and excitatory signals *λ*^−^ and *λ*^+^ respectively. The state of each cell is denoted by *q*, and it receives an inhibitory input from the state of some cell *u* which does not belong to *M*(*n*), therefore for any cell *i* Є *M*(*n*) there is an inhibitory weight w^−^(u) ≡ w^−^(u,i) > 0 from u to i.

**Figure 4:**
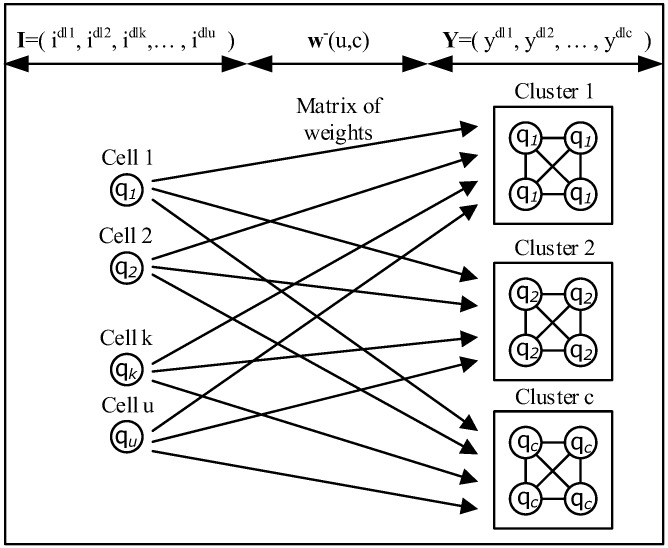
Deep Learning Cluster.

The DL Architecture is composed of *C* multiple clusters, each of which is made up of an *M*(*n*) cluster each with n hidden neurons (Figure 4). For the *c*-th such cluster, *c* = 1, …, *C*, the state of each of its identical cells is denoted by *q_c_*. In addition, there are *U* input cells which do not belong to these *C* clusters, and the state of the *u*-th cell *u* = 1, …, *U* is denoted by $\bar{q_{u}}$. The cluster network has *U* input cells and *C* clusters. The Deep Learning clusters model defines:

- **I** = (i^dl1^, i^dl2^, …, i^dlu^), *U*-dimensional vector **I** Є [0,1]*^U^* for the input state $\bar{q_{u}}$ for the cell *u*;
- **w**^−^(u,c), *U* × *C* matrix of weights from the *U* input cells to the cells in each of the *C* clusters;
- **Y** = (y^dl1^, y^dl2^, …, y^dlc^), a *C*-dimensional vector **Y** Є [0,1]*^C^* for the cell state *q_c_* for the cluster *c*.

The network learns the *U* × *C* weight matrix **w**^−^(u,c) by calculating new values of the network parameters for the input **I** and output **Y** using Gradient Descent learning algorithm which optimizes the network weight parameters **w**^−^(u,c) from a set of input-output pairs (i_u_,y_c_).

### 3.4. Deep Learning Management Clusters

**Figure 5:**
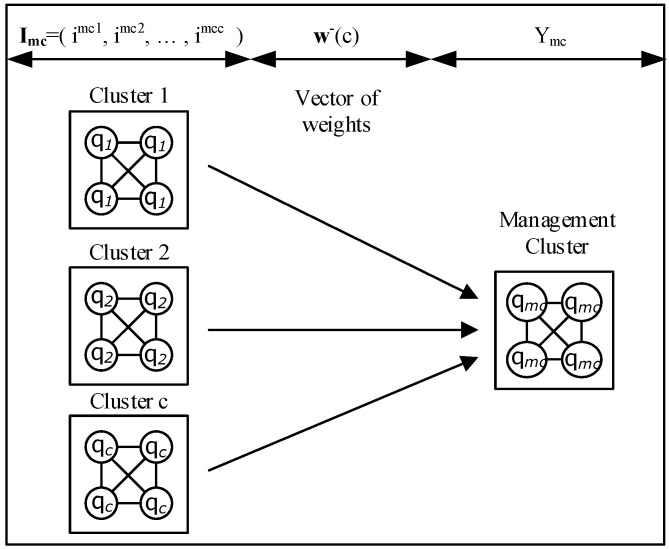
Deep Learning Management Cluster.

The Deep Learning management cluster was proposed by Serrano et al. [43]. It takes management decisions based on the inputs from different Deep Learning clusters (Figure 5); the Deep Learning Management cluster supervises the Deep Learning Clusters. The Deep Learning Management Cluster defines:

- **I_mc_** = (i^mc1^, i^mc2^, …, i^mcc^), *C*-dimensional vector **I_mc_** Є [0,1]^*C*^ for the input state $\bar{q_{c}}$ for the cluster *c*;
- **w**^−^(c), *C*-dimensional vector of weights from the *C* input clusters to the cells in the Management Cluster mc;
- Y_mc_, a scalar Y_mc_ Є [0,1], the cell state q_mc_ for the Management Cluster mc.

### 3.5. Deep Learning Cluster Structures

#### 3.5.1. Deep Learning Cluster Model

**Figure 6:**
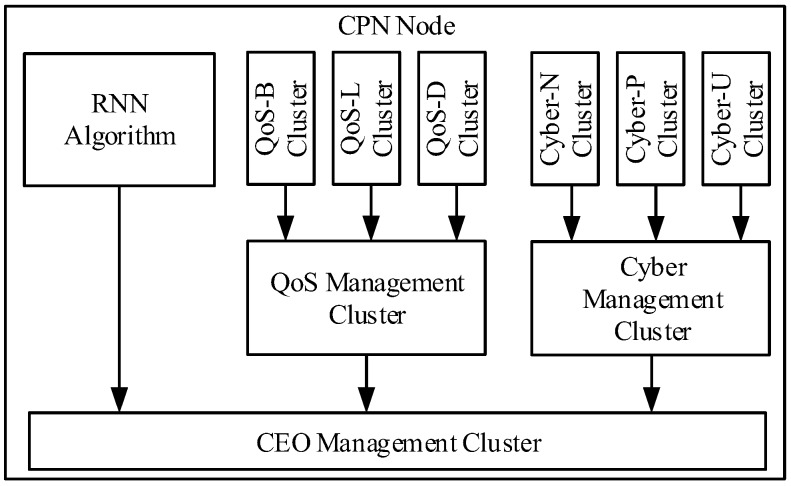
Deep Learning Cluster Structure.

The DL Cluster Structure emulates the way the brain learns and makes choices by combing different learning algorithms. The proposed model is based on the RNN Reinforcement Learning for fast local decisions and DL for long-term memory to remember network identity: QoS metrics (Delay, Loss and Bandwidth) and Cyber keys (User, Packet and Node). The addition of a layer of DL Management Clusters (QoS, Cyber and CEO) takes the final routing decision based on the inputs from the DL QoS clusters and RNN Reinforcement Learning algorithm (Figure 6). The Deep Learning Cluster Structures has been applied in the CPN for Quality of Service metrics and Cyber Security keys in Management Decisions based on packet routing and flow control.

The RNN RL Algorithm is chosen by the CEO DL Management Cluster under normal or conscious operations due to its fast and adaptable routing learning as short memory whereas DL clusters are selected under external cyber-attacks based on the long-term memory in unconsciousness operation as a safe and resilient although inefficient and inflexible routing.

The RNN RL Algorithm instantaneously updates its network weights based on the direct observations from the network; this enables its routing algorithm to take quick decisions adaptable to changes. Deep Learning algorithm adapts slowly to network changes where the proposed model applies it as a reliable and safe routing when the CPN is compromised by a Cyber-attack; it emulates the brain in a subconscious mode with long-term memory; where it takes minimum decisions for defense or survival.

#### 3.5.2. Deep Learning Clusters

DL clusters (Appendix A) learn the network identity that consists of QoS network metrics, including best routes for each QoS metric, and Cyber keys. A DL cluster is assigned to each QoS metric: Delay, Packet Loss and Bandwidth. Each QoS DL cluster learns the best-associated QoS metric with its best-associated node gates. When a node observes a better QoS route with a lower QoS metric; it learns its value and includes the gate on the first position of the QoS DL routing table.

In addition, a DL cluster is assigned per Cyber key: User, Packet and Node. The user cyber network weights authenticate the application that has transmitted the packet. The packet cyber network weights validate the packet transmitted is legitimate; this secures the network against Denial of Service attacks. The node cyber network weights authenticate the nodes within the CPN; this secures the CPN against impostor nodes. The Cyber network weights could have been assigned previously to the CPN nodes by the network administrator or the CPN nodes could have learnt them in an initialization mode. When a CPN node receives a CP; each Cyber DL cluster extracts its relevant keys and uses them as input and output values. If the quadratic error between the Cyber DL cluster output vector and the input vector is over a threshold then the CPN node considers the certificate as invalid or the CPN is under Cyber-attack.

This model defines three QoS clusters; Delay, Packet Loss and Bandwidth:

- **I_QoS_** = (i^QoS^_1_, i^QoS^_2_, …, i^QoS^_u_) a *U*-dimensional **I_QoS_** Є [0,1]*^U^* vector where i^QoS^_1_, i^QoS^_2_, and i^QoS^_u_ are the same value for each QoS type;
- **w**^−^**_QoS_**(u,c) is the *U* × *C* matrix of weights of the QoS Deep Learning Cluster;
- **Y_QoS_** = (y^QoS^_1_, y^QoS^_2_, …, y^QoS^_c_) a *C*-dimensional vector Y_QoS_ Є [0,1]*^C^* where y^QoS^_1_ is the QoS metric and y^QoS^_2_, …, y^QoS^_c_ are the node’s QoS best routing gates.

In addition, this model defines three Cyber clusters; User Packet and Node:

- **I_Cyber_** = (i^Cyber^_1_, i^Cyber^_2_, …, i^Cyber^_u_) a *U*-dimensional vector **I_Cyber_** Є [0,1]*^U^* where i^Cyber^_1_, i^Cyber^_2_, …, i^Cyber^_u_ are the Cyber keys from the CP;
- **w**^−^**_Cyber_**(u,c) is the *U* × *C* matrix of weights of the Cyber Deep Learning Cluster;
- **Y_Cyber_** = (y^Cyber^_1_, y^Cyber^_2_, …, y^Cyber^_c_) a *C*-dimensional vector **Y_Cyber_** Є [0,1]*^C^* where y^Cyber^_1_, y^Cyber^_2_, …, y^Cyber^_c_ are the Cyber keys from the DL cluster.

#### 3.5.3. Deep Learning Management Cluster

**Figure 7:**
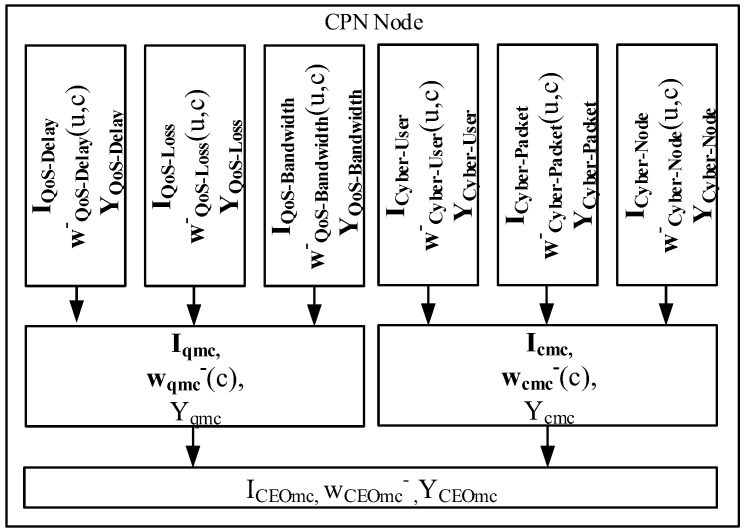
CPN node with Deep Learning clusters model.

The DL management clusters take the overall routing management decision (Figure 7). The QoS and Cyber management clusters analyze the output from their associated QoS and Cyber DL clusters respectively. If the Cyber management cluster detects a failure in the cyber certificates; the CEO management cluster routes the network Cognitive Packets as safe mode using the QoS DL clusters, otherwise, if the Cyber certificates are valid the CEO management cluster chooses the route provided by the RNN-RL routing algorithm as normal mode.

This model defines the QoS management cluster as:

- **I_qmc_** = (i^qmc^_1,_ i^qmc^_2_, … i^qmc^_c_), a *C*-dimensional vector **I_qmc_** Є [0,1]*^C^* with the values of the QoS Metrics for each QoS cluster;
- **w_qmc_**^−^(c) is the *C*-dimensional vector of weights that represents the Goal = (α_Delay_, β_Loss_, γ_Bandwidth_);
- Y_qmc_, a scalar Y_qmc_ Є [0,1] that represents the best QoS metric routing decision to be taken.

Cyber management cluster as:

- **I_cmc_** = (i^cmc^_1_, i^cmc^_2_, … i^cmc^_c_), a *C*-dimensional vector **I_cmc_** Є [0,1]*^C^* with the values of the key errors for each Cyber cluster (User, Packet, Node);
- **w_cmc_**^−^(c) is the *C*-dimensional vector of weights that represents the relevance of each Cyber Cluster;
- Y_cmc_, a scalar Y_cmc_ Є [0,1] that represents if the packet has passed the Cyber network security.

CEO management cluster as:

- I_CEOmc_, a scalar I_CEOmc_ Є [0,1] with the values of the QoS management cluster;
- w_CEOmc_^−^ a scalar w_CEOmc_^−^ Є [0,1] that represents the error of the Cyber management cluster;
- Y_CEOmc_, a scalar Y_CEOmc_ Є [0,1] that represents the final routing decision.

## 4. Implementation

**Figure 8:**
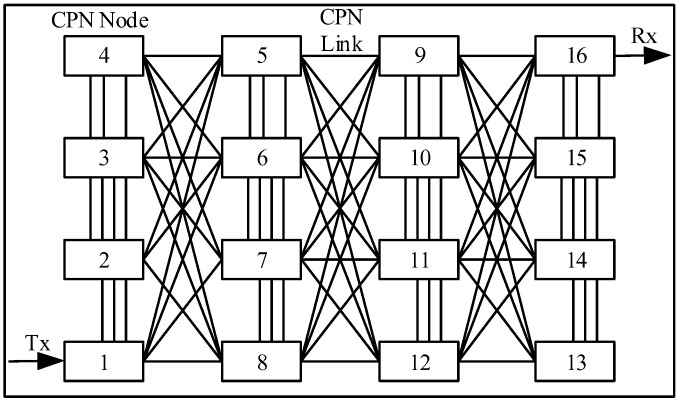
CPN Network (4 × 4 Nodes).

The Deep Learning Clusters Structure for Management Decisions is implemented in the CPN using the Network Simulator Omnet 5.0. The simulation covers several size nxn square CPNs where all the nodes in the same and adjacent layers are connected with each other. For simplicity, the simulation always considers the first node (Node 1) as the only transmitter and the last node (Node n) as the only receiver; the other nodes only participate in the routing of Cognitive Packets. An example of a 4 × 4 network is shown in Figure 8.

Each node has normalized QoS Delay, Loss and Bandwidth metrics as relative to their number; in an *n* × *n* network node *i* will have Delay: 10*i*; Loss: 5(*n* − *i*) and Bandwidth: 5 + 10*i* respectively. The approach is represented in Table 1 for a 4 × 4 network. After two Cognitive Packets are sent with a defined QoS; the QoS metric swaps between each internal node the within the same column for a 4 × 4 CPN. This model proposes to set the RNN-RL network weights with initialization packets sent at random gates.

### 4.1. Quality of Service Deep Learning Cluster

The QoS DL clusters have three input cells (*u* = 3) and three output clusters (*c* = 3). The model therefore has i^QoS-d^_1_ = 0.5; i^QoS-d^_2_ = 0.5 and i^QoS-d^_3_ = 0.5; y^QoS-d^_1_ is the best QoS Delay metric, y^QoS-d^_2_ the best QoS Delay route and y^QoS-d^_3_ the second best Delay route. The model follows a similar approach for the Loss and Bandwidth QoS DL clusters respectively. The model normalizes the inputs of the DL clusters to (0.5+ QoS Metric/1000) and (0.5+ Best Gate/100) respectively.

### 4.2. Cyber Deep Learning Cluster

The Cyber DL clusters have ten input cells (*u* = 10) and ten output clusters (*c* = 10). The key is a vector of 10 dimensions. i^Cyber-u^_u_, i^Cyber-p^_u_, i^Cyber-n^_u_ have a value between 0.1 and 0.9 with increments 0.1∆. The Cyber DL clusters network weights are trained with the value of the input the same as the output.

### 4.3. Deep Learning Management Cluster

The inputs of the Cyber management cluster are the errors provided by each Cyber DL cluster and the value of its network weights are set with the same value (0.1) therefore different cyber DL clusters have the same priority. The output Y_cmc_ is the overall Cyber quantified error decision based on a threshold. The input of the QoS management cluster are the best QoS metrics from each QoS DL cluster and the value of its networks weights corresponds to the Goal = (αDelay, βLoss, γBandwidth). The output Y_qmc_ is quantified best QoS metric decision.

The input of the CEO management cluster is the value provided by the QoS management cluster and its network weight is the value provided by the Cyber management cluster. The output is the final routing decision between the different gates provided by the RNN-RL algorithm, Delay, Loss and Bandwidth DL clusters.

## 5. Experimental Results

The DL Clusters Structure for Management Decisions has been simulated in three different *n* × *n* Cognitive Packet Network sizes, 3 × 3, 4 × 4, and 5 × 5 with different Cyber keys; QoS metrics and Goal changes to assess the routing decision-making of our proposed DL Structure. Please note that we are not evaluating the routing protocol but the routing decision.

### 5.1. Cyber Deep Learning cluster results

The different Cyber DL clusters are validated where the security keys are modified at node 1 and the cyber validation error is measured at the next node 4 once the CPs have a stable route. The keys are gradually changed; from the correct key to 0.1∆ increments applied to the different key dimensions.

The Cyber DL cluster error largely increases even only with one 0.1∆ increment (Table 2). The results are consistent between the different Cyber DL clusters. Cyber key increments have a bigger error if they are applied in the same dimension rather than split into different dimensions.

### 5.2. Quality of Service Deep Learning Cluster Results (3 × 3 Nodes)

The 3 × 3 CPN is simulated with a continuous 160 Cognitive Packet stream. The first 20 packets are used to initialize the CPN network. Goal changes after 20 packets whereas QoS metric changes 2 packets after the new Goal is selected following *T_l_* = 0.9*T*_*l*−1_ + 0.1*R* where *T_l_* is the Threshold at decision packet *l* and *R* is the Reward. The QoS DL clusters have been validated with seven different variable Goals for the same Cognitive Packet stream (Table 3).

The average error and learning algorithm iteration values for the QoS and Cyber DL clusters is shown in Table 4. The learning error of the QoS and Cyber DL Clusters is very reduced.

The number of updates in the network weights, or routing table, for the DL cluster and the RNN Reinforcement Learning is represented in Table 5.

The RNN Reinforcement Learning algorithm continuously updates its network weighs whereas the DL Cluster route only refreshes when a better route is found, however, the number of required iterations to update RNN-RL is only one whereas QoS DL clusters require approximately 160 iterations as shown in Table 4. The route decision taken by the CEO Management Cluster when the Cyber management cluster has authorized the different Cyber keys is shown in Table 6, Table 7 and Table 8 for only three different Goals as the simulation includes seven.

**Figure 9:**
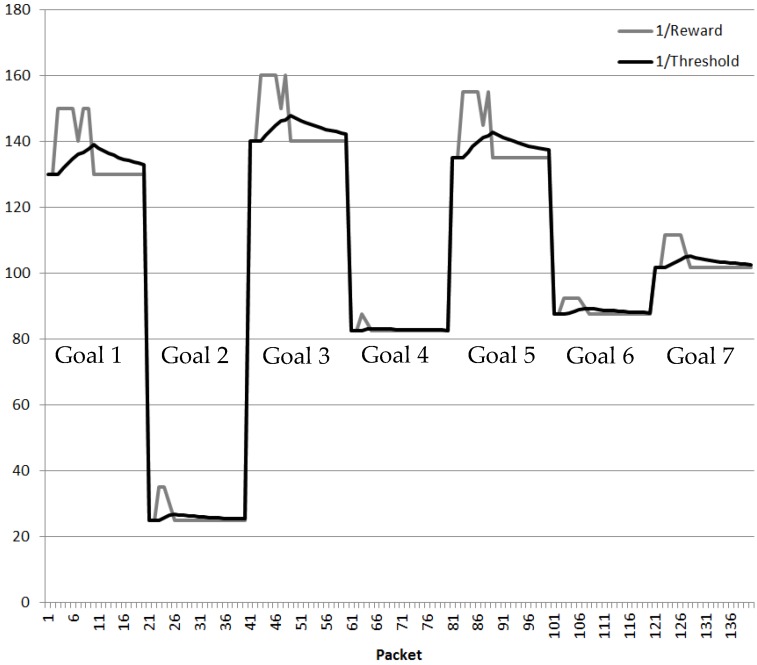
QoS Deep Learning cluster validation (3 × 3 Nodes).

The route provided by the QoS DL clusters remains unchanged due to its slow learning process until the new best route is found by the RNN-RL. The Reward and Threshold of route decision taken by the CEO Management Cluster when the Cyber management cluster has authorized the different Cyber keys is shown in Figure 9 for the seven different Goals. When the new best route is discovered; the CPN Threshold adapts gradually to the original value.

### 5.3. Deep Learning Management Cluster Results (3 × 3 Nodes)

The DL Management Clusters (Cyber, QoS and CEO) on this section are validated under two different Cyber Security scenarios; ∆ = 0: normal operation and ∆ = 0.1: CPN under Cyber-attack. Three different strategic Cognitive Packets (CP 30, CP 85 and CP 148) are chosen for the 3 × 3 CPN validation with different Goals. Results are shown in Table 9.

### 5.4. Quality of Service Deep Learning Cluster Results (4 × 4 Nodes)

The 4 × 4 CPN is simulated with a continuous 380 Cognitive Packet stream. The first 100 packets are used to initialize the CPN network. Goal changes after 40 packets whereas QoS metric changes 2 packets after the new Goal is selected following *T_l_* = 0.99*T*_*l*−1_ + 0.01*R* where *T* is the Threshold at decision packet *l* and *R* is the Reward. The QoS DL clusters have been validated with seven different variable Goals for the same Cognitive Packet stream (Table 10).

The average error and learning algorithm iteration values for the QoS and Cyber DL clusters is shown in Table 11.

The number of updates in the network weights or routing table for the DL cluster and the RNN Reinforcement Learning is shown in Table 12.

The number of iterations to update RNN-RL is only one whereas DL clusters require approximately 150 iterations as shown in Table 11. The route decision taken by the CEO Management Cluster when the Cyber management cluster has authorized the different Cyber keys are shown in Table 13, for the first Goal only.

**Figure 10:**
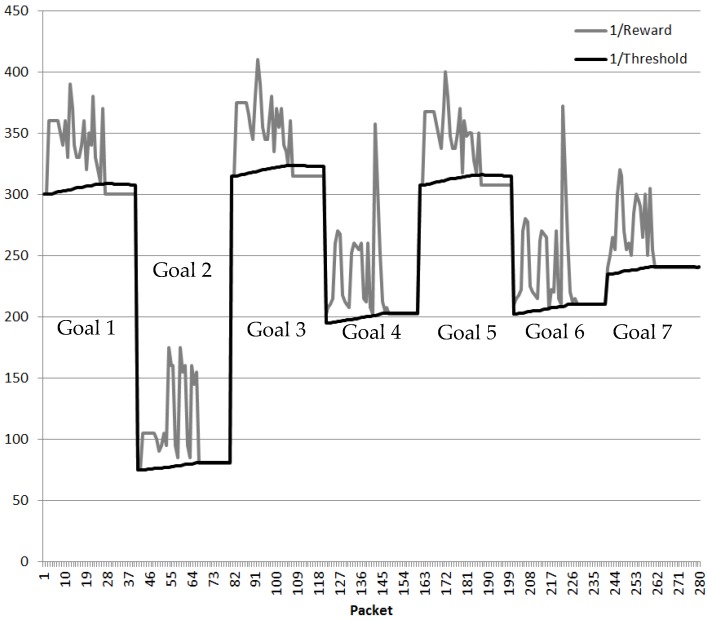
QoS DL Cluster validation (4 × 4 Nodes).

The results provided by the 4 × 4 CPN are similar to the 3 × 3 CPN. The first two packets follow the best route whereas the third packet acknowledges the QoS metrics have changed. RNN-RL finds the optimum route after Cognitive Packets explore the network and DL learns the route a Cognitive Packet after. The Reward and Threshold of route decision taken by the CEO Management Cluster when the Cyber management cluster has authorized the different Cyber keys is shown in Figure 10 for the seven different Goals. When the new best route is discovered; the CPN Threshold adapts gradually to the original value.

### 5.5. Deep Learning Management Cluster Results (4 × 4 Nodes)

The results provided by the DL management cluster confirm the proposed model. The correct quantification of the DL management cluster cell states and the selection of the accurate thresholds are fundamental to take relevant optimum decisions. Three different strategic Cognitive Packets are chosen (CP 107, CP 228 and CP 341) for the 4 × 4 CPN validation, where each one has a different Goal. Results for the two different Cyber Security scenarios; ∆ = 0: normal operation and ∆ = 0.1: CPN under Cyber-attack are shown in Table 14.

### 5.6. Quality of Service Deep Learning Cluster Results (5 × 5 Nodes)

The 5 × 5 CPN is simulated with a continuous 1550 Cognitive Packet stream. The first 1500 packets are used to initialize the CPN network with a single 1.0 × Delay Goal after 50 packets whereas QoS metric changes 2 packets after the Goal is selected following *T_l_* = 0.999*T*_*l*−1_ + 0.01*R*. The QoS DL clusters have been validated with only one Goal for the same Cognitive Packet stream (Table 15).

The average error and learning algorithm iteration values for the QoS and Cyber Deep Learning clusters is shown in Table 16.

The number of updates in the network weights, or Routing Table for the DL cluster and the RNN Reinforcement Learning is represented in Table 17.

**Figure 11:**
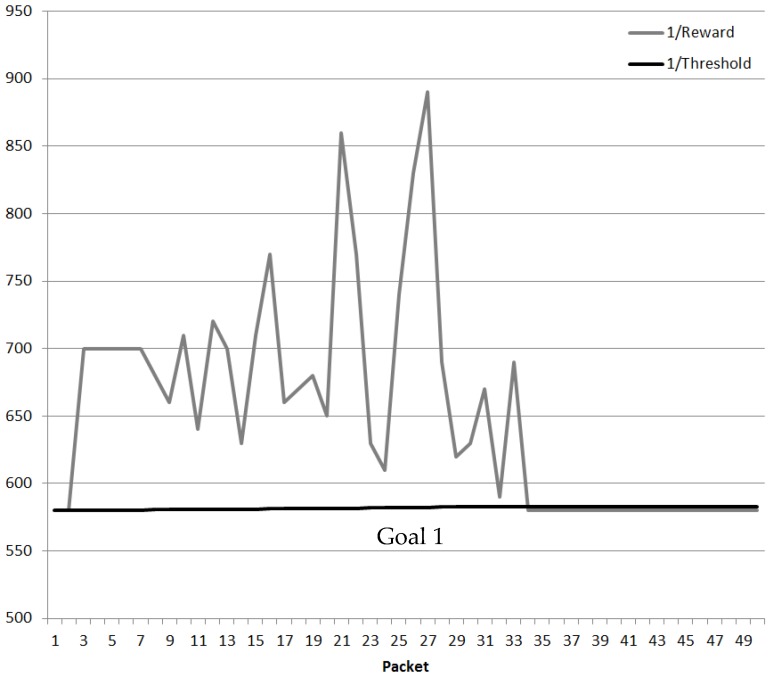
QoS DL Cluster validation (5 × 5 Nodes).

The Network keeps sending Cognitive Packets until the value of the 1/Reward is lesser than the 1/Threshold. When the new best route is discovered as shown in Figure 11; the CPN Threshold adapts gradually to the original value.

### 5.7. Deep Learning Management Cluster Results (5 × 5 Nodes)

Results for the two different Cyber Security scenarios; ∆ = 0: normal operation and ∆ = 0.1: CPN under Cyber-attack are shown in Table 18. For the 5 × 5 CPN, the results of the DL Management cluster are consistent with the previous results, the DL management cluster adapts to network changes and provides the optimum route based on the current network conditions.

## 6. Conclusions

This paper has presented a Deep Learning Cluster Structure for Management Decisions. The proposed hierarchical decision model has been validated in the Cognitive Packet Network with three configurations: small size 3 × 3, medium size 4 × 4 and large size 5 × 5 with one, two and three layers of decision respectively. The addition of Deep Learning clusters specialized in different functions (Cyber, QoS, and Management) provides a flexible approach similar to how our brain performs; Deep Learning clusters are able to adapt and being assigned where more routing, computing and memory resources are required.

The RNN Reinforcement Learning algorithm adapts very quickly to variable QoS changes with fast decisions in short-term memory; whereas Deep Learning is slow to adapt to QoS changes as it learns from the RNN-DL algorithm and stores routing information in long-term memory. The CEO management cluster takes the right routing decisions based on the inputs from the QoS and Cyber Management Clusters. This allows the CPN to use a safe route in case of Cyber-attack, or a fast route under normal conditions. Future work will expand the validation gradually up to very large-scale networks (100 nodes, 8 decision layers).

## Figures and Tables

**Table 1:** QoS Values (4 × 4 Nodes).

**Node 4 Initial—Final**	**Node 5 Initial—Final**	**Node 9 Initial—Final**	**Node 16 Initial—Final**
Delay: 40–40	Delay: 50–80	Delay: 90–120	Delay: 160–160
Loss: 65–65	Loss: 60–45	Loss: 40–25	Loss: 05–05
Bandwidth: 45–45	Bandwidth: 55–85	Bandwidth: 95–125	Bandwidth: 165–165
**Node 3 Initial—Final**	**Node 6 Initial—Final**	**Node 10 Initial—Final**	**Node 15 Initial—Final**
Delay: 30–30	Delay: 60–70	Delay: 100–110	Delay: 150–150
Loss: 70–70	Loss: 55–50	Loss: 35–30	Loss: 10–10
Bandwidth: 35–35	Bandwidth: 65–75	Bandwidth: 105–115	Bandwidth:155–155
**Node 2 Initial—Final**	**Node 7 Initial—Final**	**Node 11 Initial—Final**	**Node 14 Initial—Final**
Delay: 20–20	Delay: 70–60	Delay: 110–100	Delay: 140–140
Loss: 75–75	Loss: 50–55	Loss: 30–35	Loss: 15–15
Bandwidth: 25–25	Bandwidth: 75–65	Bandwidth: 115–105	Bandwidth: 145–145
**Node 1 Initial—Final**	**Node 8 Initial—Final**	**Node 12 Initial—Final**	**Node 13 Initial—Final**
Delay: 10–10	Delay: 80–50	Delay: 120–90	Delay: 130–130
Loss: 80–80	Loss: 45–60	Loss: 25–40	Loss: 20–20
Bandwidth: 15–15	Bandwidth: 85–55	Bandwidth: 125–95	Bandwidth: 135–135

**Table 2:** Cyber Deep Learning Cluster Validation.

Dimension	∆ = 0.0	∆ = 0.1	∆ = 0.2	∆ = 0.3	∆ = 0.4
1	9.7500 × 10^−11^	0.0102	0.0409	0.0921	0.1638
2	9.7537 × 10^−11^	0.0213	0.0851	0.1915	0.3406
3	9.7537 × 10^−11^	0.0326	0.1305	0.2938	0.5226
4	9.7537 × 10^−11^	0.0451	0.1806	0.4067	0.7238
5	9.7537 × 10^−11^	0.0576	0.2306	0.5195	0.9249
6	9.7537 × 10^−11^	0.0715	0.2867	0.6465	1.1519
7	9.7537 × 10^−11^	0.0851	0.3414	0.7703	1.3732
8	9.7537 × 10^−11^	0.1006	0.4038	0.9119	1.6273
9	9.7537 × 10^−11^	0.1153	0.4633	1.0470	1.8698
10	9.7537 × 10^−11^	0.1323	0.5321	1.2038	2.1526

**Table 3:** QoS Deep Learning Cluster Validation (3 × 3 Nodes)—Simulation Parameters.

Packet	Goal Number	Goal Description	QoS
001–020	-	Network Initialization Packets	
021–022	1	1 × Delay	Initial Values
023–040	1	1 × Delay	Final Values
041–042	2	1 × Loss	Initial Values
043–060	2	1 × Loss	Final Values
061–062	3	1 × Bandwidth	Initial Values
063–080	3	1 × Bandwidth	Final Values
081–082	4	0.5 × Delay + 0.5 × Loss	Initial Values
083–100	4	0.5 × Delay + 0.5 × Loss	Final Values
101–102	5	0.5 × Delay + 0.5 × Bandwidth	Initial Values
103–120	5	0.5 × Delay + 0.5 × Bandwidth	Final Values
121–122	6	0.5 × Loss + 0.5 × Bandwidth	Initial Values
123–140	6	0.5 × Loss + 0.5 × Bandwidth	Final Values
141–142	7	0.3 × Delay + 0.3 × Loss + 0.3 × Bandwidth	Initial Values
143–160	7	0.3 × Delay + 0.3 × Loss + 0.3 × Bandwidth	Final Values

**Table 4:** Deep Learning Cluster Validation (3 × 3 Nodes).

Cyber DL Cluster	Error	Iteration	QoS DL Cluster	Error	Iteration
Cyber User	6.96 × 10^−10^	58	QoS Delay	9.59 × 10^−10^	163.67
Cyber Packet	7.34 × 10^−10^	108	QoS Loss	9.16 × 10^−10^	163.14
Cyber Node	9.94 × 10^−10^	1162.33	QoS Bandwidth	9.16 × 10^−10^	135.33

**Table 5:** Deep Learning Cluster vs RNN-RL (3 × 3 Nodes).

Updates	RNN-RL	QoS Delay	QoS Loss	QoS Bandwidth
Initialization	0	4	1	3
CP 021-160	140	9	1	9

**Table 6:** Goal: 1 × Delay (3 × 3 Nodes).

Packet	RNN-RL Route	DL Route	Best Route	Goal 1/Reward	1/Threshold
021	1-4-9	1-4-9	1-4-9	130.00	130.00
022	1-4-9	1-4-9	1-4-9	130.00	130.00
023	1-4-9	1-4-9	1-6-9	150.00	130.00
024	1-4-9	1-4-9	1-6-9	150.00	131.76
025	1-4-9	1-4-9	1-6-9	150.00	133.38
026	1-4-9	1-4-9	1-6-9	150.00	134.87
027	1-5-9	1-4-9	1-6-9	140.00	136.25
028	1-4-9	1-4-9	1-6-9	150.00	136.61
029	1-2-6-9	1-4-9	1-6-9	150.00	137.84
030	1-6-9	1-4-9	1-6-9	130.00	138.97
031	1-6-9	1-6-9	1-6-9	130.00	138.02
040	1-6-9	1-6-9	1-6-9	130.00	132.99

**Table 7:** Goal: 0.5 × Delay + 0.5 × Loss (3 × 3 Nodes).

Packet	RNN-RL Route	DL Route	Best Route	Goal 1/Reward	1/Threshold
081	1-4-9	1-4-9	1-4-9	82.50	82.50
082	1-4-9	1-4-9	1-4-9	82.50	82.50
083	1-4-9	1-4-9	1-6-9	87.50	82.50
084	1-5-9	1-4-9	1-6-9	85.00	82.97
085	1-6-9	1-4-9	1-6-9	82.50	83.17
086	1-6-9	1-6-9	1-6-9	82.50	83.10
087	1-6-9	1-6-9	1-6-9	82.50	83.04
088	1-6-9	1-6-9	1-6-9	82.50	82.99
089	1-6-9	1-6-9	1-6-9	82.50	82.94
090	1-6-9	1-6-9	1-6-9	82.50	82.90
091	1-6-9	1-6-9	1-6-9	82.50	82.86
100	1-6-9	1-6-9	1-6-9	82.50	82.64

**Table 8:** Goal: 0.3 × Delay + 0.3 × Loss + 0.3 × Bandwidth (3 × 3 Nodes).

Packet	RNN-RL Route	DL Route	Best Route	Goal 1/Reward	1/Threshold
141	1-4-9	1-4-9	1-4-9	101.66	101.66
142	1-4-9	1-4-9	1-4-9	101.66	101.66
143	1-4-9	1-4-9	1-6-9	111.66	101.66
144	1-4-9	1-4-9	1-6-9	111.66	102.58
145	1-4-9	1-4-9	1-6-9	111.66	103.42
146	1-4-9	1-4-9	1-6-9	111.66	104.18
147	1-5-9	1-4-9	1-6-9	106.66	104.89
148	1-6-9	1-4-9	1-6-9	101.66	105.06
149	1-6-9	1-6-9	1-6-9	101.66	104.71
150	1-6-9	1-6-9	1-6-9	101.66	104.40
151	1-6-9	1-6-9	1-6-9	101.66	104.12
160	1-6-9	1-6-9	1-6-9	101.66	102.60

**Table 9:** DL Management Cluster Validation (3 × 3 Nodes).

Variable	Cognitive Packet: 30 G: 1.0 × D + 0.0 × L + 0.0 × B		Cognitive Packet: 85 G: 0.5 × D + 0.5 × L + 0.0 × B		Cognitive Packet: 148 G: 0.3 × D + 0.3 × L + 0.3 × B	
Cyber Attack	∆ = 0.0	∆ = 0.1	∆ = 0.0	∆ = 0.1	∆ = 0.0	∆ = 0.1
Cyber I_cmc_	5 × 10^−11^	3.4 × 10^−4^	5 × 10^−11^	3.4 × 10^−4^	5 × 10^−11^	3.4 × 10^−4^
Cyber Y_cmc_	0.9994	0.9969	0.9994	0.9969	0.9994	0.9969
QoS-Delay I_qmc_	0.6300	0.6300	0.3150	0.3150	0.2100	0.2100
QoS-Loss I_qmc_	0.0000	0.0000	0.2625	0.2625	0.1750	0.1750
QoS-Band I_qmc_	0.0000	0.0000	0.0000	0.0000	0.2133	0.2133
QoS-Delay Y_qmc_	0.1765	0.1765	0.3000	0.3000	0.3913	0.3913
QoS-Loss Y_qmc_	0.9994	0.9994	0.3396	0.3396	0.4354	0.4354
QoS- Band Y_qmc_	0.9994	0.9994	0.9994	0.9994	0.3875	0.3875
CEO I_CEOmc_	0.1000	0.1000	0.1000	0.1000	0.9000	0.9000
CEO w_CEOmc_^−^(c)	0.0000	0.9999	0.0000	0.9999	0.0000	0.9999
CEO Y_CEOmc_	0.9994	0.5746	0.9994	0.5746	0.9994	0.1305
Routing Decision	RNN-DL Gate-4 Node 6	DL-Delay Gate-2 Node 4	RNN-DL Gate-4 Node 6	DL-Delay Gate-2 Node 4	RNN-DL Gate-4 Node 6	DL-Band Gate-2 Node 4

**Table 10:** QoS Deep Learning Cluster Validation (4 × 4 Nodes) —Simulation Parameters.

Cognitive Packet	Goal Number	Goal Description	QoS Metric
000–100	-	Network Initialization Cognitive Packets	
001–002	1	1.0 × Delay + 0.0 × Loss + 0.0 × Bandwidth	Initial Values
003–040	1	1.0 × Delay + 0.0 × Loss + 0.0 × Bandwidth	Final Values
041–042	2	0.0 × Delay + 1.0 × Loss + 0.0 × Bandwidth	Initial Values
043–080	2	0.0 × Delay + 1.0 × Loss + 0.0 × Bandwidth	Final Values
081–082	3	0.0 × Delay + 0.0 × Loss + 1.0 × Bandwidth	Initial Values
083–120	3	0.0 × Delay + 0.0 × Loss + 1.0 × Bandwidth	Final Values
121–122	4	0.5 × Delay + 0.5 × Loss + 0.0 × Bandwidth	Initial Values
123–160	4	0.5 × Delay + 0.5 × Loss + 0.0 × Bandwidth	Final Values
161–162	5	0.5 × Delay + 0.0 × Loss + 0.5 × Bandwidth	Initial Values
163–200	5	0.5 × Delay + 0.0 × Loss + 0.5 × Bandwidth	Final Values
201–202	6	0.0 × Delay + 0.5 × Loss + 0.5 × Bandwidth	Initial Values
203–240	6	0.0 × Delay + 0.5 × Loss + 0.5 × Bandwidth	Final Values
241–242	7	0.3 × Delay + 0 × 3Loss + 0.3 × Bandwidth	Initial Values
243–280	7	0.3 × Delay + 0 × 3Loss + 0.3 × Bandwidth	Final Values

**Table 11:** Deep Learning Cluster Validation (4 × 4 Nodes).

Cyber DL Cluster	Error	Iteration	QoS DL Cluster	Error	Iteration
Cyber User	6.96 × 10^−10^	58.00	QoS Delay	9.34 × 10^−10^	158.67
Cyber Packet	7.34 × 10^−10^	108.00	QoS Loss	9.22 × 10^−10^	152.07
Cyber Node	9.93 × 10^−10^	1017.87	QoS Bandwidth	8.83 × 10^−10^	127.60

**Table 12:** Deep Learning Cluster vs. RNN-RL (4 × 4 Nodes).

Updates	RNN-RL	QoS Delay	QoS Loss	QoS Bandwidth
Initialization	0	8	6	7
CP 001-280	280	9	4	9

**Table 13:** Goal: 1 × Delay (4 × 4 Nodes).

Packet	RNN-RL Route	DL Route	Best Route	Goal 1/Reward	1/Threshold
001	1-5-9-16	1-5-9-16	1-5-9-16	300.00	300.00
002	1-5-9-16	1-5-9-16	1-5-9-16	300.00	300.00
003	1-5-9-16	1-5-9-16	1-8-12-16	360.00	300.00
004	1-5-9-16	1-5-9-16	1-8-12-16	360.00	300.50
005	1-5-9-16	1-5-9-16	1-8-12-16	360.00	301.00
006	1-5-9-16	1-5-9-16	1-8-12-16	360.00	301.49
007	1-5-9-16	1-5-9-16	1-8-12-16	360.00	301.98
008	1-6-9-16	1-5-9-16	1-8-12-16	350.00	302.47
009	1-7-9-16	1-5-9-16	1-8-12-16	340.00	302.88
010	1-2-6-10-16	1-5-9-16	1-8-12-16	360.00	303.21
011	1-8-9-16	1-5-9-16	1-8-12-16	330.00	303.69
012	1-4-5-10-16	1-5-9-16	1-8-12-16	390.00	303.93
013	1-3-5-11-16	1-5-9-16	1-8-12-16	370.00	304.61
014	1-5-11-16	1-5-9-16	1-8-12-16	340.00	305.15
015	1-6-11-16	1-5-9-16	1-8-12-16	330.00	305.46
016	1-7-10-16	1-5-9-16	1-8-12-16	330.00	305.69
017	1-2-7-11-16	1-5-9-16	1-8-12-16	340.00	305.91
018	1-4-6-12-16	1-5-9-16	1-8-12-16	360.00	306.22
019	1-8-10-16	1-5-9-16	1-8-12-16	320.00	306.68
020	1-3-6-12-16	1-5-9-16	1-8-12-16	350.00	306.80
021	1-5-11-16	1-5-9-16	1-8-12-16	340.00	307.18
022	1-4-3-7-12-16	1-5-9-16	1-8-12-16	380.00	307.48
023	1-2-8-11-16	1-5-9-16	1-8-12-16	330.00	308.07
024	1-6-12-15	1-5-9-16	1-8-12-16	320.00	308.27
025	1-7-12-15	1-5-9-16	1-8-12-16	310.00	308.39
026	1-3-4-8-12-16	1-5-9-16	1-8-12-16	370.00	308.40
027	1-8-12-16	1-5-9-16	1-8-12-16	300.00	308.92
028	1-8-12-16	1-8-12-16	1-8-12-16	300.00	308.82
029	1-8-12-16	1-8-12-16	1-8-12-16	300.00	308.73
030	1-8-12-16	1-8-12-16	1-8-12-16	300.00	308.64
040	1-8-12-16	1-8-12-16	1-8-12-16	300.00	307.80

**Table 14:** DL Management Cluster Validation (4 × 4 Nodes).

Variable	Cognitive Packet: 107 G: 1.0 × D + 0.0 × L + 0.0 × B		Cognitive Packet: 228 G: 0.5 × D + 0.5 × L + 0.0 × B		Cognitive Packet: 341 G: 0.3 × D + 0.3 × L + 0.3 × B	
Cyber Attack	∆ = 0.0	∆ = 0.1	∆ = 0.0	∆ = 0.1	∆ = 0.0	∆ = 0.1
Cyber I_cmc_	5 × 10^−11^	3.4 × 10^−4^	5 × 10^−11^	3.4 × 10^−4^	5 × 10^−11^	3.4 × 10^−4^
Cyber Y_cmc_	0.9994	0.9969	0.9994	0.9969	0.9994	0.9969
QoS-Delay I_qmc_	0.8000	0.8000	0.4000	0.4000	0.2666	0.2666
QoS-Loss I_qmc_	0.0000	0.0000	0.2875	0.2875	0.1916	0.1916
QoS-Band I_qmc_	0.0000	0.0000	0.0000	0.0000	0.2716	0.2716
QoS-Delay Y_qmc_	0.1444	0.1444	0.2523	0.2523	0.3361	0.3361
QoS-Loss Y_qmc_	0.9994	0.9994	0.3195	0.3195	0.4132	0.4132
QoS- Band Y_qmc_	0.9994	0.9994	0.9994	0.9994	0.3319	0.3319
CEO I_CEOmc_	0.1000	0.1000	0.1000	0.1000	0.9000	0.9000
CEO w_CEOmc_^−^(c)	0.0000	0.9999	0.0000	0.9999	0.0000	0.9999
CEO Y_CEOmc_	0.9994	0.5746	0.9994	0.5746	0.9994	0.1305
Routing Decision	RNN-DL Gate-6 Node 8	DL-Delay Gate-3 Node 5	RNN-DL Gate-6 Node 8	DL-Delay Gate-6 Node 8	RNN-DL Gate-6 Node 8	DL-Band Gate-3 Node 5

**Table 15:** QoS Deep Learning Cluster Validation—Simulation Parameters (5 × 5 Nodes).

Cognitive Packet	Goal Number	Goal Description	QoS Metric
0000–1500	-	Network Initialization Cognitive Packets	
001–002	1	1.0 × Delay + 0.0 × Loss + 0.0 × Bandwidth	Initial Values
003–050	1	1.0 × Delay + 0.0 × Loss + 0.0 × Bandwidth	Final Values

**Table 16:** Deep Learning Cluster Validation (5 × 5 Nodes).

Cyber DL Cluster	Error	Iteration	QoS DL Cluster	Error	Iteration
Cyber User	7.56 × 10^−13^	62	QoS Delay	9.4 × 10^−13^	221.11
Cyber Packet	8.60 × 10^−13^	125	QoS Loss	9.30 × 10^−13^	182.40
Cyber Node	9.91 × 10^−13^	2128.68	QoS Bandwidth	9.30 × 10^−13^	200.71

**Table 17:** Deep Learning Cluster vs RNN-RL (5 × 5 Nodes).

Updates	RNN-RL	QoS Delay	QoS Loss	QoS Bandwidth
Initialization	0	8	20	7
CP 001-050	50	1	0	0

**Table 18:** DL Management Cluster Validation (5 × 5 Nodes).

Variable	Cognitive Packet: 034 G: 1.0 × D + 0.0 × L + 0.0 × B	
Cyber Attack	∆ = 0.0	∆ = 0.1
Cyber I_cmc_	5.14 × 10^−14^	3.47 × 10^−4^
Cyber Y_cmc_	0.9994	0.9969
QoS-Delay I_qmc_	0.5590	0.5590
QoS-Loss I_qmc_	0.0000	0.0000
QoS-Band I_qmc_	0.0000	0.0000
QoS-Delay Y_qmc_	0.1945	0.1945
QoS-Loss Y_qmc_	0.9994	0.9994
QoS- Band Y_qmc_	0.9994	0.9994
CEO I_CEOmc_	0.1000	0.1000
CEO w_CEOmc_^−^(c)	0.0000	0.9999
CEO Y_CEOmc_	0.9994	0.5746
Routing Decision	RNN-RL Gate-8 Node 10	DL-Delay Gate-4 Node 6

**Figure A1:**
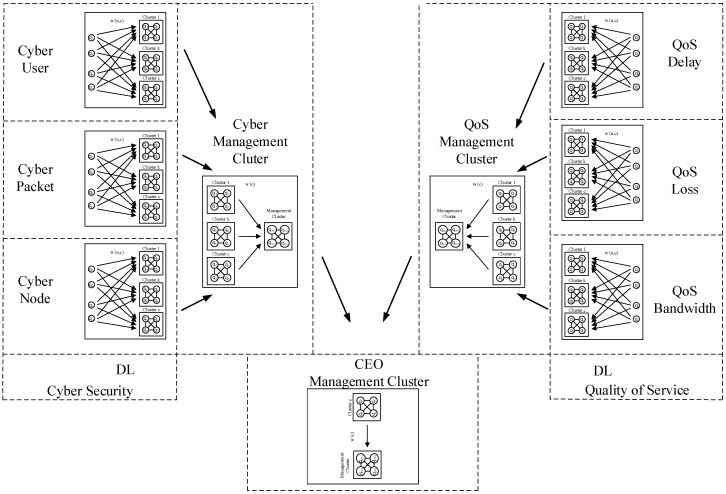
Deep Learning Cluster Structures for Management Decisions—Neural Schematic.
